# Development of a risk-tailored approach and dashboard for efficient management and monitoring of investigator-initiated trials

**DOI:** 10.1186/s12874-023-01902-y

**Published:** 2023-04-05

**Authors:** Katharina Klatte, Suvitha Subramaniam, Pascal Benkert, Alexandra Schulz, Klaus Ehrlich, Astrid Rösler, Mieke Deschodt, Thomas Fabbro, Christiane Pauli-Magnus, Matthias Briel

**Affiliations:** 1 Department of Clinical Research, University Hospital Basel and University of Basel, Spitalstrasse 12, Basel, CH- 4031 Switzerland; 2 Department of Public Health & Primary Care, KU Leuven, Leuven, Belgium; 3 Competence Centre of Nursing, University Hospitals Leuven, Leuven, Belgium; 4 Department of Health Research Methods, Evidence, and Impact, McMaster University, Hamilton, ON Canada

**Keywords:** Clinical trial, Trial management, Risk-tailored monitoring, Trial dashboard

## Abstract

**Background:**

Most randomized controlled trials (RCTs) in the academic setting have limited resources for clinical trial management and monitoring. Inefficient conduct of trials was identified as an important source of waste even in well-designed studies. Thoroughly identifying trial-specific risks to enable focussing of monitoring and management efforts on these critical areas during trial conduct may allow for the timely initiation of corrective action and to improve the efficiency of trial conduct. We developed a risk-tailored approach with an initial risk assessment of an individual trial that informs the compilation of monitoring and management procedures in a trial dashboard.

**Methods:**

We performed a literature review to identify risk indicators and trial monitoring approaches followed by a contextual analysis involving local, national and international stakeholders. Based on this work we developed a risk-tailored management approach with integrated monitoring for RCTs and including a visualizing trial dashboard. We piloted the approach and refined it in an iterative process based on feedback from stakeholders and performed formal user testing with investigators and staff of two clinical trials.

**Results:**

The developed risk assessment comprises four domains (patient safety and rights, overall trial management, intervention management, trial data). An accompanying manual provides rationales and detailed instructions for the risk assessment. We programmed two trial dashboards tailored to one medical and one surgical RCT to manage identified trial risks based on daily exports of accumulating trial data. We made the code for a generic dashboard available on GitHub that can be adapted to individual trials.

**Conclusions:**

The presented trial management approach with integrated monitoring enables user-friendly, continuous checking of critical elements of trial conduct to support trial teams in the academic setting. Further work is needed in order to show effectiveness of the dashboard in terms of safe trial conduct and successful completion of clinical trials.

**Supplementary Information:**

The online version contains supplementary material available at 10.1186/s12874-023-01902-y.

## Introduction

Randomized controlled trials (RCTs) are the gold standard for assessing the effects of medical interventions. However, they are typically resource intense and pose various organisational challenges [1–3]. Inefficient management and monitoring of RCTs have been identified as an important source of waste [1–5]. Monitoring efforts are traditionally quite generic and extensive, [6–8] but problems such as slow participant recruitment, considerable losses to follow-up, or poor data quality are often recognized too late during trial conduct delaying necessary adjustments of processes or the protocol. In addition, resources for clinical trial monitoring and management are usually scarce in the academic setting and sophisticated commercial solutions can be costly [9, 10].

Organisational challenges and critical factors jeopardizing trial integrity and quality may vary considerably across trials; therefore, a risk assessment conducted prior to trial initiation or at certain intervals during trial conduct may yield different risk profiles for individual trials. Trial monitoring protects the safety and rights of participants, ensures data are accurate, complete and verifiable, and that the trial follows the principles of good clinical practice [11, 12]. Currently recommended risk-based trial monitoring allows for an adaptation of the monitoring intensity according to an initial risk assessment of a trial and has been developed to reduce resource intense onsite visits with source data verification for non-high-risk trials [1–3, 13–15, 16, 17. However, this approach typically does not consider individual risk profiles of RCTs, but rather classifies trials by generic risk categories [16]. To accommodate individual trial risks, a monitoring strategy may include several components such as centralized monitoring (evaluation of accumulated trial data performed in a timely manner at a central location), onsite monitoring (performed at investigator sites with source data verification and review of protocol-specified processes), or remote monitoring (same tasks as onsite monitoring but performed away from investigator sites) [17, 18, 19].

Trial management should provide for smooth and reliable trial procedures including participant recruitment, randomisation, intervention application, data collection, and data cleaning [20, 21]. Data cleaning and checking of recruitment and retention rates, for instance, need to be performed in a timely fashion, so that corrective measures can be taken early on and detrimental effects on the trial can be avoided [22]. Trial monitoring is most effective when performed on cleaned data, because incorrect processes may be missed due to poor data quality and monitoring efforts are wasted on individual data errors. Therefore, trial management and monitoring ideally are integrated tasks that make use of accumulating data during trial conduct, i.e. continuously keeping oversight of complex study processes and performing centralized data monitoring [23–25].

The objective of this project was to develop a risk-tailored approach that integrated trial management and monitoring in investigator-initiated RCTs. We closely collaborated with relevant stakeholders (trial coordinators, principal investigators, data managers, trial monitors, statisticians) to create a user-friendly dashboard that efficiently visualizes data on critical processes of individual trials.

## Methods

### Overview of research process

**Fig. 1:**
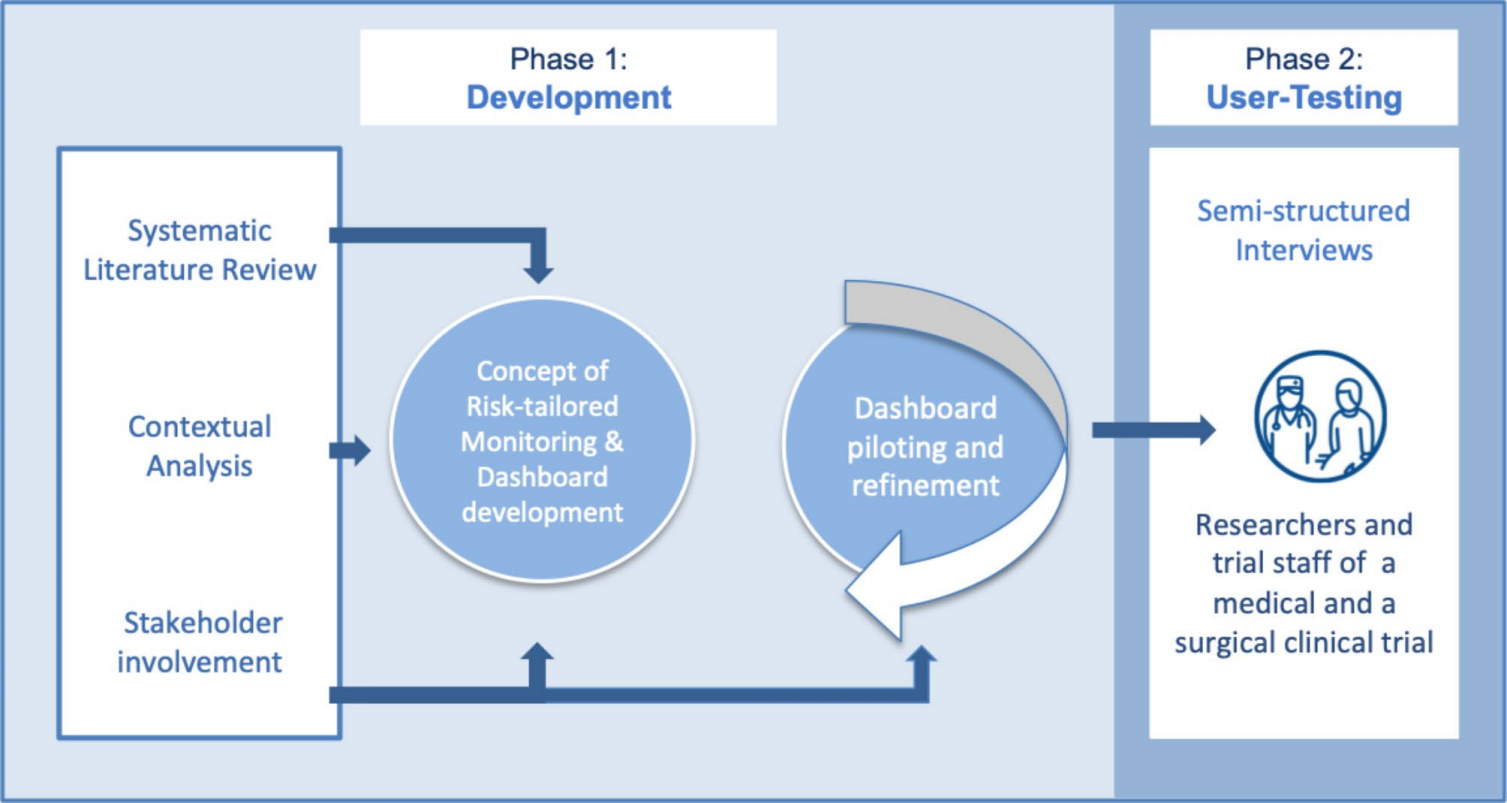
Overview of the two phases of the development and user-testing of the risk-tailored approach and trial dashboard

In the first phase of this user-centred project, [26] we developed a concept of a risk-tailored trial monitoring and management approach with corresponding trial dashboard (Fig. 1). We anticipated users to be primarily trial managers, principal investigators, and trial monitors. The development involved relevant stakeholder groups and was based on the results of systematic literature reviews on existing monitoring strategies, [17] and a contextual analysis to identify current practices and needs of anticipated users. The concept and dashboard were piloted and refined in an iterative process involving different end users and other stakeholder groups. In the second phase, we performed formal user testing of the developed risk assessment and dashboard. Experiences of investigators and trial staff of one medical and one surgical investigator-initiated RCT were gathered using semi-structured interviews to further refine the concept and dashboard.

### Setting

Before the introduction of the new concept, a risk assessment was routinely performed by the monitoring team to assess the extent of the monitoring needed for the trial according to the ADAMON criteria. This approach allowed the rough classification of trials into the categories low, medium, or high risk [27]. The new risk assessment incorporates many more factors related to the study specific conduct including challenges in the study management. It is not meant to categorize trials and adjust the extent of monitoring based on the category. The trial teams included in our project were not involved in other pre-trial risk assessments. Both trial teams assessing the benefits of the risk assessment and dashboard tool had started participant recruitment and data collection before the implementation of the new tool and, thus, compared it to the situation without structured risk assessment and tool support.”

#### Systematic literature review

To identify and structure components for the initial risk assessment of individual trials, we systematically searched for published risk assessment approaches and risk indicators used to support trial oversight and to identify centres in need for support. We considered different components and qualitative evidence from process evaluations of tested monitoring strategies summarized in a previously conducted systematic review [17]. We further considered the guideline of the European Clinical Research Infrastructure Network (Ecrin) [16] and the risk assessment guideline developed by the Swiss Clinical Trial Organization [28], TransCelerate metrics [29, 30], Whitham metrics [31], and the trial specific metrics used by the Medical Research Council (MRC) Clinical Trials Unit (CTU) at University College London (UCL) Trial specific metrics [32]. Results from this literature review are summarized in Supplementary Table 1.

#### Stakeholder involvement

We set up a local, multidisciplinary working group including end users and representatives of different stakeholder groups within the Department of Clinical Research (DKF) and associated research groups at the University Hospital Basel. At this local level, we involved members from the Data Science and Data Management Teams of the DKF experienced in central monitoring, R shiny applications, dashboard development, database structures and exports; we involved trial monitors with experience in on-site and remote monitoring, knowledge of study site structures and processes; study coordinators and investigators experienced in managing RCTs. Stakeholder meetings with all members of these groups provided an additional opportunity for feedback and exchange of information on the risk assessment and dashboard development as well as on the application strategy. In order to get input from a national group of stakeholders in Switzerland, we contacted the national platform of the Swiss Clinical Trial Organisation for trial monitoring. Finally, we gathered experiences from international methodological research groups and UK-based CTUs using risk-based approaches or study dashboards to support trial conduct. The different activities with stakeholders at all levels are summarized in Supplementary Table 2. We extracted information from protocols of meetings and interviews and summarized the output in Supplementary Table 3.

### Contextual analysis

Gathering contextual input from various end users and the above-mentioned stakeholders guided the development of the risk-tailored approach and helped to determine relevant domains and applications to be considered in the initial risk assessment. We structured the identified stakeholder needs into content related factors such as the inclusion of the follow-up visits into the risk assessment, and design related factors such as the suggested separation of severity and likelihood in the assessment or the colour code for the status of queries visualized in the dashboard (Supplementary Table 3). In terms of content of the risk assessment, it became clear, for instance, that the assessment covers a wide spectrum of risks applicable to a large variety of RCTs. The design of the risk assessment guide should support the intuitive assessment by different end user groups (monitors, study managers, principal investigators). The study dashboard should reflect the outcome of the risk assessment and the design of the dashboard should enable an efficient navigation within the routine study procedure by end-users. The findings of the contextual analysis are summarized in Supplementary Table 3.

#### Development and piloting of the concept and dashboard

Based on the systematically reviewed literature, our contextual analysis and stakeholder input, we drafted a generic risk-assessment template. We then created trial-specific dashboards for a medical and a surgical multicentre trial that differed in their risk profile, but both comprised complex study procedures and data collection. The risk-tailored approach continued to evolve as we gathered contextual information, detected gaps in the assessment procedure, and identified critical components of study management. We developed R code to extract data values from exported data tables of the trial database secuTrial and summarized, compared, and calculated relevant information to create pathways for the identified risks. The output of these operations was then visualized in the trial dashboard. The piloting and refinement was an iterative process incorporating repeated feedback from the end-users and the stakeholder representatives in the project group on dashboard content, structure, user-friendly interface, and visualization of critical study data.

#### User testing

The aim of the user testing was to identify challenges in the routine use of the dashboard experienced by different user groups. Each of the six users (i.e. 2 trial managers, 2 monitors, 2 principal investigators) received a detailed manual of the features and operation mode of the study dashboard.

We interviewed users 6–12 weeks after using the study dashboard in daily trial routine. We followed a semi-structured interview guide, which allowed for expansion on topics that emerged during the interview. All interviews took approximately 30 min. The interviewer (KK) transcribed the recorded interviews and extracted suggestions for improvement. We then updated the trial dashboard based on the feedback of the users and provided the adapted version for further use and evaluation.

## Results

The final concept consisted of the following three steps: trial-specific risk assessment prior to study start, selection and development of data-based pathways to address identified risks, and visualization of pathways output in a trial dashboard.

### Trial-specific risk assessment

The trial-specific risk assessment comprised four domains (participant safety and rights, overall study management, device/medication management, study data), and each domain contained several risk elements (Table 1**).** To better assess if these elements are critical for a specific trial and which trial components are at particular risk, we determined trial assets and corresponding risk scenarios. Trial assets are conditions essential for the successful and proper conduct of a trial, e.g. visits must be scheduled and take place in the required timeframe, Serious Adverse Events (SAEs) have to be reported on time and need to be closely followed over the whole study conduct. If a trial includes many follow-up visits over a long follow-up time and assessments have to take place in a very narrow time window, this asset would be considered at risk (example shown in Table 2, Part A). Other assets, for example SAE reporting and oversight, are essential for all clinical trials and, thus, are considered as a risk that applies to all trials (marked in red, Example shown in Table 2, Part B). The identified risks are then analysed in terms of severity and likelihood. For example, if many follow-up visits need to be coordinated but the time window of the endpoint assessment is wide the severity is rated as less critical. The likelihood is highly influenced by the experience of the trial team and participating centres with similar trials, training and experience of all involved staff members, and the resources available for the study.

The complete list of assets, as well as the corresponding risk scenarios, is provided in the full risk assessment in Supplementary Table 4. We suggest that the risk assessment is done by an experienced trial manager (e.g. from a trials support unit) supported by a trial monitor, a clinical expert, and the principal investigator. The first risk assessment should be performed before the start of the trial based on the study protocol, Case Report Forms (CRFs), the planned and actual budget of the study, expected recruitment rates for all participating centres, information on the trial intervention, and information about planned study staff (see Appendix for detailed **Manual**).

**Table 1:** Domains and their attributed risk elements

Domain	Risk Elements
Participant Safety and Rights	Informed consent AE/SAE reporting and documentation Inclusion/exclusion
Overall Study Management	Recruitment Retention Study procedures and endpoint assessment (e.g. bio sampling, imaging quality) Participant schedule (e.g. timeframe of visits) AE/SAE management
Device/ Medication Management	Administration Accountability/ storage
Study Data	Data quality – completeness, consistency, timeliness Documentation/ storage

Abbreviations: AE, adverse event; SAE, serious adverse event

**Table 2:** Example of assets and risk scenarios for risk elements in the domain Overall Study Management (Part A) and Participant Safety and Rights (Part B). Assets that apply to all trials are marked in red

A)			
Domain	Risk element	Asset	Risk scenario
Overall Study Management	**Participant Schedule**	Visits/Phone calls must be within the given Timeframe	(A) Time point of visit is critical for the endpoint assessment of the study
			**(**B) Large number of visits are difficult to organize and coordinate between centres and patients
**B)**			
Domain	Risk element	Asset	Risk scenario
Participant Safety and Rights	**SAE/AE**	SAE have to be reported and documented correctly in the required timeframe	Complexity of CRF or missing SOPs for SAE Reporting leads to (A) Incorrect documentation and (B) Delayed reporting of SAEs

Abbreviations: CRF, case report form; SOPs, standard operating procedures; SAE, serious adverse events

### Pathways to manage identified risks

**Fig. 2:**
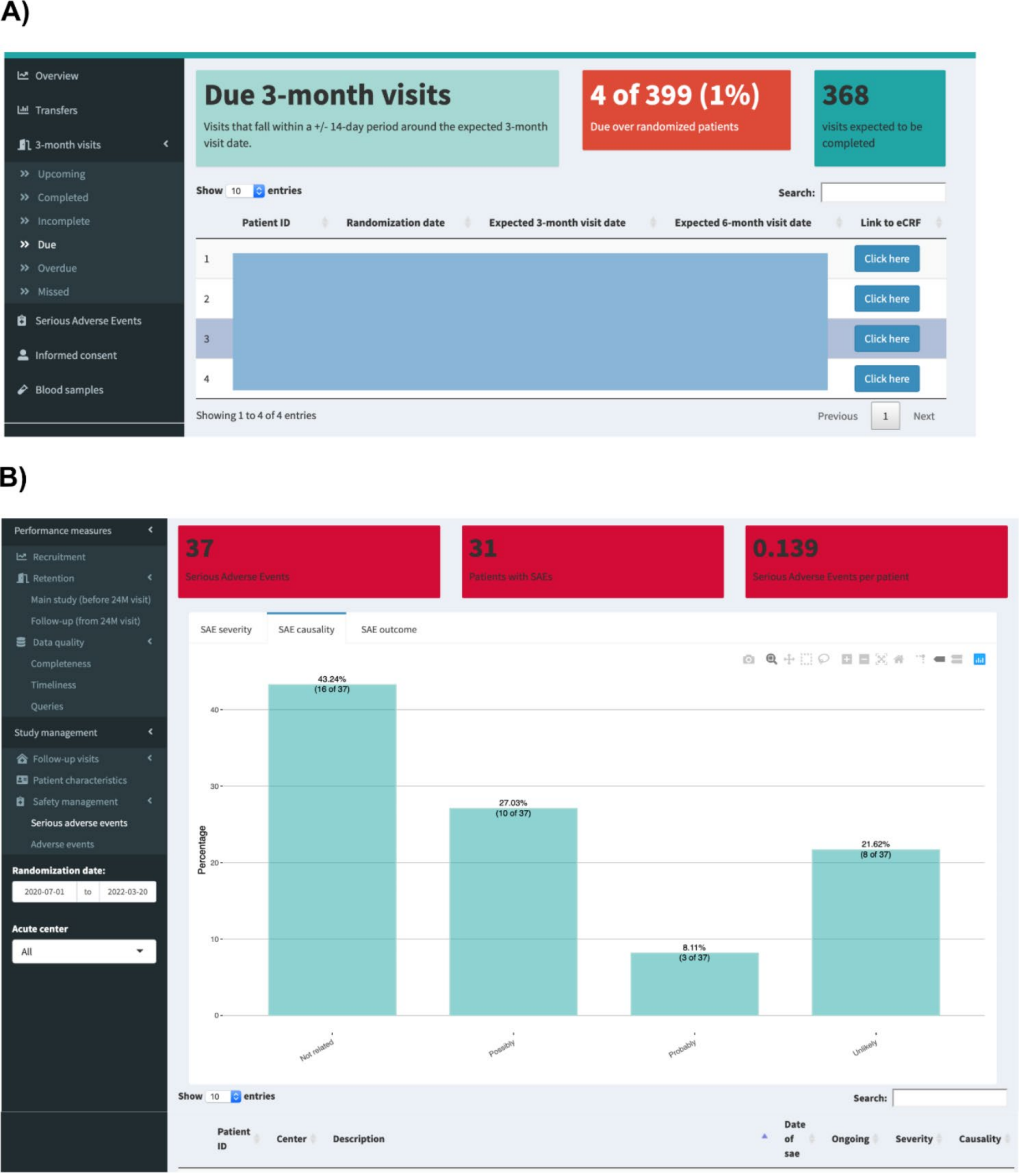
Dashboard screenshots of the Visits tab, sub-tab “Due visits” (Panel **A**), and the Safety management tab, sub-tab “Serious adverse events” (Panel **B**)

In order to continuously manage identified risks, we created pathways that eventually allowed for tailored visualization of accumulating trial data and implemented action at suitable time intervals (e.g., email reminders, staff overviews) in a study dashboard. The operations applied to the exported data tables via R code are dependent on the specific information needed to provide a clear oversight on identified risk elements. The code is structured into modules that contain the operations of all pathways visualized in one dashboard tab (e.g. SAE management). For example, the module SAE contains operations that count the number of SAEs, determine the number of patients with SAE and calculate the ratio SAEs per patient randomized. In addition, information like severity, causality and outcome are extracted from the SAE form data table and percentages of value options (e.g. SAE outcome: Continuing, Resolved without sequel, Resolved with sequel, others) are calculated and graphically displayed (Fig. 2, Panel A and B). The developed study dashboards contain tabs that visualize the output of created pathways reflecting identified study-specific risks. These tabs are based on the R modules containing the pathways as well as the code required for a clear visual presentation (value boxes, graphs, lists). When pilot testing our risk assessment guide, it became apparent that some risks apply to almost all trials (marked in red in the full risk assessment Supplementary Table 4). The management of these risks is, thus, based on tabs classified as “generic” in the study dashboard, while other, more seldom and study-specific risks are considered in “optional” tabs (Table 3). The content of generic tabs can also be adapted depending on, for instance, the complexity or time point of outcome assessment in a trial. The generic dashboard template is freely available on GitHub (https://github.com/CTU-Basel/viewTrial).

### Visualization of data based pathways

The output of the pathways is visualized in the corresponding tabs in the study dashboard. The arrangement of the tabs within the study dashboard can be determined by study teams; a division into study management related tabs and oversight/study progress tabs may provide a better overview for the different user groups (principal investigator, study manager, and trial monitor). The main tabs can also contain sub-tabs. For example, the number of due visits is displayed under the visits tab in the sub-category “due visits”. In this context, the definitions of due, overdue, and missed visits are dependent on the specific timeframes of the study protocol. Total numbers are provided as well as a list of the patient ID and a direct link to the corresponding eCRF in the database (Fig. 2, Panel A). Each tab or sub-tab can represent several pathway outputs displayed in form of value boxes, graphical presentations, or lists of relevant patients. For example, the SAE management tab provides an overview on SAE prevalence in boxes, and in additional panels the user can switch between the graphical representation of SAE severity, causality, and outcome. Additionally, a list of patients with SAE is provided below, displaying information on SAE status (e.g. ongoing/closed) and a short description of the event (Fig. 2, Panel B). The information is provided for the overall study, including all randomized patients as numbers and percentages in boxes, while graphs differentiating between centres are provided to better assess which centres are in need for support in a certain aspect of the study conduct. In addition, the dashboard allows filtering for specific centres and time ranges of interest or choosing particular study visits from drop down menus to provide users with more detailed information (see Supplementary Fig. 1 for an example). The output of the pathways visualized in the dashboard is based on a daily export of trial data and, thus, includes up-to-date information on randomised patients and entered data. The generic and some of the optional tabs are listed in Table 3. Examples of the tabs from the two study dashboards are provided in Supplementary Figs. 2–5. The generic dashboard is accessible via GitHub and generic data is provided to test the different code modules behind each tab (examples provided in Supplementary Figs. 6 and 7).

**Table 3:** Structure and content of dashboard tabs

Domain	Risk Elements	Example Tabs	Content of Tab	Functionality/Purpose	Generic/Optional
Participant Safety and Rights	Informed consent	**Informed consent**	In case of a re-consent this tab can provide an overview of patients patients who have previously not been able to give consent themselves	To ensure patient rights and support of re-consent process through site-specific reminders, list of patients that still need a re-consent.	Optional
	AE/SAE reporting and documentation	**AE/SAE**	Provides an overview of timeliness and completeness of AE/SAE entries	To ensure that all AE/SAE forms are complete and that the date of first entry is within the required reporting timeframe	Generic
	Inclusion/exclusion	**Safety**	In case of safety-relevant inclusion or exclusion criteria, a verification of relevant information available in the database can provide additional security (e.g. blood pressure has to be within a certain range – check for the entry of blood pressure in the database)	To provide the option for additional checks for inclusion/ exclusion criteria besides the marked list of criteria in the eCRF	Optional
Overall Study Management	Recruitment	**Recruitment**	Recruitment trajectories for expected and actual recruitment in total and per centre (Supplementary Fig. 2)	To monitor the progress of participant recruitment enabling early action in case of slow recruitment.	Generic
		**Patient Characteristics**	Relevant patient characteristics are summarized and presented (e.g. gender, age, background of treatment)	To inform the study team on the accuracy of inclusion/exclusion criteria and provide an overview of the sample population in terms of relevant characteristics	Generic
	Retention	**Retention**	Patients who have ended the study resulting in missing outcome data, reasons for leaving the study, kind of data collected before study end (Primary outcome data available) (Supplementary Fig. 3)	To monitor the progress of participant retention, consider reasons for ending study in recruitment. Time point of ending the study important for amount of data analysable.	Generic
	Study procedures and endpoint assessment	**Bio sampling (e.g. blood samples)**	Overview of samples taken and availability of sample results	To support sample management in terms of localization and status of bio sample. Important for biomarker determination.	Optional
		**Imaging quality**	Automated and visual verification of imaging data quality, e.g., for MRI or CT	To enable early adjustments in case of low quality imaging data and ensure that the imaging data is analysable.	Optional
	Participant schedule:	**Follow-up visits**	Overview of follow-up visits with a particular focus on visits where primary outcome data is collected. **(**Fig. 2, **Panel A**)	To assist in integrating follow-up visits on time into the daily clinical routine might be difficult for trial sites. Support through reminders for due visits can be initiated through the dashboard.	Optional
	AE/SAE management	**Safety management (SAEs, AEs)**	The Safety tab provides an overview of SAEs and AEs that have been reported in the study and information on severity and outcome of SAEs/AEs (Fig. 2, Panel B)	To estimate potential safety issues (e.g. SAEs occurring more often in one study arm, number of SAEs in total, number of patients with SAE)	Generic
Device/ Medication Management	Administration Accountability/ storage	**Medication**	Overview of medication consumption based on number of patients and their current position in the medication plan per protocol and comparison with IMP stock at sites	To assist in the managing of IMP stock overview and enable reminders for restocking	Optional
Study Data	Data quality – completeness, consistency, timeliness Documentation/ storage	**Data Quality**	Completeness of forms (Primary end point, secondary endpoint, SAE/AE forms) Timeliness of data entry, Number of queries, status of queries (open, resolved) (Supplementary Figs. 4,5)	To increase awareness of items missing in the database Trial sites may have different challenges when integrating a trial in their daily clinical routine and therefore need support in different aspects of the study conduct. Completeness and timeliness of data entry as well as query management constitute indicators for need of support. Query status helps the study monitor to decide which centre needs more assistance/ on-site visit.	Generic

Abbreviations: AEs, adverse events; CT, computerized tomography ;IMP, investigational medicinal product; MRI, magnetic resonance imaging; SAEs, serious adverse events

### User testing

The user testing of our study dashboards provided positive feedback in terms of improved study oversight and facilitated conduct. Trial monitors and study staff agreed that the initial risk assessment was beneficial, because it increased the awareness of critical processes in the collection of outcome data, enabling corrective measures at an early time point, e.g. adaptation of database structures. A clear benefit perceived by all user groups was the more frequent and improved communication with trial sites; sites were better prepared for remote or on-site monitoring visits, because many issues were recognized and solved in advance. In addition, users made several suggestions for further elements to be included in the dashboard. A detailed summary of the results from the user testing is provided in Supplementary Table 5.

## Discussion

Using a systematic approach involving relevant stakeholder groups, we developed a concept of risk-tailored trial monitoring and management that focuses on the identification and control of trial specific risks during trial conduct. The continuous evaluation of most important risks provides important information about the study progress, e.g. in terms of recruitment, endpoint assessment, as well as in terms of data management and data quality, e.g. CRF completion, timeliness of follow-up visits. Completeness of essential data points as the basis for analysable patient data is continuously evaluated and trial monitors and study managers maintain an overview of visit timeframes, SAE reporting, and query management.

### Strengths and limitations

Strengths of our study are the systematic and structured process of development of the risk assessment and the trial dashboard, which included the involvement of all local stakeholder groups and the performance of a comprehensive contextual analysis. In addition, the development was based on prior evidence gathered through systematic literature searches and exchange with international stakeholder groups. Directly involving end users in developing and evaluating the usability of our tool may facilitate the implementation process, promote wider adoption, maintain involvement, and increase user satisfaction with the concept as well as the tool [33]. Providing an R code repository for other study teams that can be adapted and applied to differently structured databases, constitutes a software-independent, affordable approach for the limited budget of investigator-initiated trials.

Our study has the following limitations: First, we performed user testing in two ongoing RCTs only, and, thus, the spectrum of feedback may have been limited and may compromise the extrapolation of mentioned benefits and disadvantages to other trials. Both RCTs had already started participant recruitment when the dashboard was implemented. This allowed for a qualitative comparison of management and monitoring processes without and with the dashboard tool in place. However, it will be crucial to subsequently evaluate the impact and value of the study dashboard during the entire course of a clinical trial. Since both RCTs are still ongoing, we could not evaluate the impact of the tool on participant safety and overall trial success, including the percentage of analysable data, at the end of a trial. Lastly, we have not yet evaluated any cost-effectiveness of our developed approach, e.g. assessing whether the dashboard has the potential to reduce monitoring and management hours needed to ensure a safe and successful trial conduct. While some users felt that our dashboard would only be worthwhile for multicentre trials, others found that the costs of providing a study dashboard will always depend on the needs and preferences of the study team and the complexity of the study.

### Comparison with similar studies and frameworks

Following the recommendations of the Clinical Trials Transformation Initiative (CTTI), effective and efficient monitoring and management needs to first determine what matters for a specific trial and focus on areas of highest risk for generating errors that matter [34, 35]. With our risk assessment guide and the study dashboard we address the need for this focus and provide a tool that supports the continuous oversight of the quality of the trial conduct.

Dashboards that visualize time-dependent parameters have recently met a growing acceptance in medical and administrative health care settings [36–43]. Dashboards have been introduced to support various aspects of clinical trials, including web applications for eligibility screening and overview of the enrolment progress [41], web-based support of recruitment management and communication; [42] graphical summaries and diagrams of the progress of patient accrual and form completion [43], feedback on data completeness by using a traffic light system [44], and automated reports of data compliance, protocol adherence and safety [45]. These available dashboards typically focus on specific elements of trial conduct and communication with trial sites; however, our dashboard provides a comprehensive overview of all elements of a trial identified as critical. In addition, tables and graphical representations are often limited to certain time intervals [41]. The daily export of trial data providing up-to-date trial information is part of the core idea of our approach as it enables immediate actions and improves communication with site staff.

Various methods for assessing the risk of non-conform trial conduct at trial sites including central statistical monitoring have been introduced in the academic setting with increasing prevalence [46]. Most methods use statistical testing of all or a subset of trial data items to compare sites and identify atypical trial centres. While many methods focus on the detection of data errors and fraud, [47] triggered monitoring is frequently used to direct on-site monitoring to atypical trial sites [46]. In our approach components of central data evaluations are used to assess whether actions are required constituting some sort of triggered intervention. However, the data evaluation is not based on statistical testing, it is rather an assessment of trial progress (recruitment, retention), management challenges, and conform data collection progress. It is also not intended to categorize trials and predetermine the extent of on-site monitoring [48]. Our concept focuses on directing attention to the most critical areas of a trial and should help to minimize and tailor on-site monitoring.

Several commercial solutions supporting the overall trial conduct in various aspects are readily available [9, 49–53], but for investigator-initiated trials with tight budgets such software packages typically remain unaffordable. We wanted to provide a comprehensive and affordable option for investigator-initiated trials that can be adapted to individual needs and preferences and further developed by the research community. Therefore, we transparently present all details of the structured risk assessment and manual as well as the generic code for our dashboard in publicly accessible repositories via GitHub. We invite users to report difficulties or suggestions for improvement for consideration in future modifications of the generic dashboard via GitHub.

### Implications

Besides the emphasis on the feasibility and design of clinical trials, measures to increase the efficiency of clinical trial conduct are needed [54]. Current challenges include premature discontinuation of a significant proportion of clinical trials, and inflated costs mainly due to delayed recruitment and organisational issues [54]. We propose a comprehensive approach integrating management and monitoring of a clinical trial into one risk management tool supporting the conduct of investigator-initiated trials.

Overseeing the progress of a trial in each centre based on up-to-date information, provides the opportunity for trial monitors to prioritize centres for on-site visits or remote interactions, tailor their action to the specific issues of a centre, and guide decisions on where resources and training is needed the most. In addition, providing automated reminders for upcoming visits or sampling, overview of investigational medicinal product supply, overview of patients who need a re-consent, overview of ongoing SAEs, etc. could increase the efficiency of the trial management processes. The tool further provides the opportunity to improve the overall communication between the study team and trial sites and may increase motivation through the involvement of sites in the trial progress and the option to compliment active participation in the trial. The dashboard tool is intended to address site-level monitoring, trial-wide monitoring, and finding per-patient issues. Feedback from the user testing also revealed a positive perception of study managers and investigators to improved data quality visible in the dashboard: “If incomplete is empty, I am at ease.

The impact of this tool is largely dependent on the successful implementation into clinical trial practice. The perception of benefits and opportunities by stakeholders and end-users have been collected while the effectiveness of the tool in terms of analysable data collected, timeline of recruitment, conformity of SAE/Adverse Event (AE) reporting and documentation, support of the overall study management still have to be evaluated.

The next step is now to implement the risk assessment as a routine step in the joint planning of clinical trials with the respective study teams. The timely generation of a dashboard on the basis of the generic template and further study-specific risks has to be organized. Strategies to further evaluate this implementation process as well as the effectiveness of this new approach in studies of different design and structure have to be developed. As an implementation outcome, the amount of studies taking advantage of the study dashboard in relation to the studies for which a dashboard was recommended could be assessed along with the frequency of risk assessments performed per trial. The effectiveness of the concept of risk assessment and dashboard tool will be evaluated based on structured feedback from study teams on their experience and quantitative measures of the trial, e.g. proportion of analysable patients/data at the end of the trial. These evaluations will provide more information on the feasibility of study-specific dashboards supporting trial monitoring and management in the heterogeneous field of clinical trials.

## Conclusion

In summary, the presented risk-assessment guide and dashboard tool provide a systematically developed and user-tested instrument for the risk-tailored support of trial monitoring and trial management. Feedback from the user testing of the instrument revealed many benefits for the involved stakeholder groups. However, the effectiveness of the dashboard in terms of a safe trial conduct and overall support for a successful completion of clinical trials needs to be further evaluated.

## Electronic supplementary material

Below is the link to the electronic supplementary material.

Supplementary Material 1

Supplementary Material 2

## Data Availability

We provide R modules as the basis for the dashboard development on GitHub (https://github.com/CTU-Basel/viewTrial). Qualitative data that supported the development of the risk assessment and study dashboard is provided in the supplementary material.
