# Supplementary material for: Development of a risk-tailored approach and dashboard for efficient management and monitoring of investigator-initiated trials
Source: BMC Med Res Methodol. 2023 Apr 5;23:84. doi: 10.1186/s12874-023-01902-y (PMC10074803; doi:10.1186/s12874-023-01902-y)
Supplement: Supplementary file 1 — Supplementary Material 1 [file 12874_2023_1902_MOESM1_ESM.doc]

**Supplementary Material**

**Supplementary Table 1:** Summary of risk indicators identified through systematic literature review

| **Domain** | **Risk indicators** |
| --- | --- |
| Recruitment | - Total number of patients enrolled at a site / the recruitment target set for the trial at this site - Total number of patients who consented/ total number of patients eligible for the trial |
| Retention | - Number of patients lost to follow-up or who withdrew from the study - Reasons for losses to follow-up/withdrawals |
| Data Quality | - Systematic errors - Abnormal trend in data, specific data item always missing - CRF completion - Average delta of visit date and data entry (CRF completion) for each centre compared to the average of all centres - Percentage of missing data items (Total number of fields available for data entry that are missed or queried / Total number of fields available for data entry) - Entry Management - Number of queries - Data query response rate - Number of overdue queries - Percentage of patients with open queries - Discrepancies between SAE event and day of reporting (more than 7 days) |
| Follow-up visits | - Are visits taking place in the required timeframe - Number of visits overdue - Number of visits missed (out of timeframe stated in study protocol) |
| Informed Consent and Eligibility | - Number of patients with informed consent date/ Number of patients randomized - Delta between consent date and randomization date - All eligibility criteria fulfilled based on protocol (If not covered by CRF rules) |

**Supplementary Table 2: Overview of stakeholder activities supporting the contextual analysis**

| **Stakeholder activity** | **Participants involved** | **N** | **Mode of stakeholder activities** | **Purpose of stakeholder activities** |
| --- | --- | --- | --- | --- |
| **Local project group meetings** | Representatives from 5 different groups: Data science, Data management, Monitoring, Study coordinators, principle investigators | 40 meetings between May 2019 and February 2021, with the number of participants ranging from 5-7 persons | In-person meetings | - To help the project team stay informed on project progress; - Identify relevant barriers; - Inform stakeholders and discuss their input and concerns related to various project components. |
| **Interviews with local Data management/Data science experts** | Data management and data science team members | 1 meeting each with 6 experts | In-person interviews | - To identify current practices and, work that has already been done in terms of central monitoring, - To identify needs and suggestions for improvement |
| **Interviews with local Monitoring team** | Monitoring team members | 1-2 meetings with each monitor – 4 members | In-person interviews | - To discuss current practice - To identify needs and suggestions for improvement |
| **Department of Clinical Research team meeting** | Department of Clinical Research (all divisions including the Data Science, Data management, Monitoring) | 2 meetings with 35-40 persons | Presentation | - To verify if all needs and concerns of the stakeholder groups have been considered in the concept development |
| **Meetings with local Principal investigators of clinical trials** | 2 Principal investigators of ongoing trials at the University Hospital Basel | Meeting once a month | In-person meeting | - To discuss needs and suggestions with principle investigators: What would help them in terms of management support and performance overview |
| **National SCTO monitoring Platform** | SCTO monitoring platform members | One meeting in Bern, email contact | In-person meeting, email correspondence | - To get input on the concept and discuss needs and suggestions with monitoring teams in Switzerland. |
| **International Clinical Trials Methodology Conference** | International Methodology Research Community | 3 day conference in Brighton with short oral & poster presentation and informal meetings | Presentation & In-person meeting | - To discuss the preliminary results of our literature review - To learn about evaluated risk indicators for trial and site performance. |
| **International collaboration with trial monitoring experts at the MRC Clinical Trials Unit at UCL** | 2 Senior statisticians from the MRC CTU at UCL | 2 meetings, email correspondence over 2 years | In-person meetings, email correspondence | - Exchange about ongoing refinement of risk indicators, - To learn about central monitoring of clinical trials established in the UK |
| **International collaboration with monitoring dashboard developers in Aberdeen** | Senior statisticians | 1 phone call, email correspondence over 3 months | Telephone call, email correspondence | - Exchange about study dashboard development and content |

Abbreviations: SCTO, Swiss Clinical Trial Organisation; MRC UCL, Medical Research Council of the University College London

**Supplementary Table 3: Overview of stakeholder input as part of the contextual analysis**

| **Step of Development** | **Content** | **Structure and Design** |
| --- | --- | --- |
| Risk assessment | - Include patient safety as well as procedural risks endangering successful trial completion - Include the management of participant schedule per protocol (e.g. timeframe of visits) - Include Data collection and storage related items - Include handling of Investigational Medicinal Product handling - Include safety management (e.g. SAE reporting, status) - Include complex informed consent processes (Re-consent) - Consider complex sampling or imaging during conduct - Take into account additional factors like experience of staff, budget | - Structured into four domains of Participant Safety and Rights, Overall Study Management, Device/Medication Management, Study Data - Structured into risk elements (e.g. Participant Schedule) and assets (a standard requirement that provides the basis for safety and accuracy of a clinical trial, e.g. visits have to take place in the required timeframe) - Provide possible risk scenarios to better apply the assessment to a study - Document rationale for rating - Assess severity and likelihood of risks |
| Study Dashboard | - Need for standardized central data monitoring (**Supplementary Table 2:** Summary of current practice) - A strong need for assistance and overview of management elements essential for study conduct (e.g. overview of follow-up visits done, recruitment curves). - A need to assess compare study progress in each site - Visualize ongoing SAEs, status of SAEs, timely reporting of SAEs - Visualize form completeness of primary outcome - Visualize patients who need a re-consent - Include status of queries - A need for site-specific information based on the entered study data to assist and guide the on-site monitoring visits - Trigger (red value boxes) for immediate phone call or email reminder to enable early resolution of a problem independent of an on-site visit - Differentiate between participants ending the study with or without the primary outcome data collected - Show analysis for reasons of ending the study (retention) | - Drop-down menu to be able to choose single visits - Colour code status of queries should be equal to database - Colour code for problems to be addressed immediately should be red - Listing the patient for whom a correction/action is needed and a link to the eCRF - Provide the option to choose patients in a specific randomization time-frame (e.g. during Covid-Pandemic) - Option to choose specific centres - Retention before and after primary outcome reached |

Abbreviations: IMP, Investigational Medicinal Product; SAE, Serious Adverse Event; eCRF, electronic Case Report Form

**Supplementary Table 4:** Full risk assessment guide. Generic content that applies to all trials is marked in red. In order to make the risk assessment operational, refer to the manual.

| Domain | Risk element | Asset | Risk scenario | Facilitator* | Likely | Critical | Rationale for Rating |
| --- | --- | --- | --- | --- | --- | --- | --- |
| **Participant Safety and Rights** | Informed consent | Condition/Characteristics of subject population and complexity of informed consent process must be considered | (A) Subject population is vulnerable (emergency situation, children, patient not able to consent) |  |  |  |  |
|  |  |  | (B) Multiple informed consent processes lead to delayed, incorrect informed consent process (e.g. pre-screening, sub-studies, Re-consent in case of next-of-kin consent) |  |  |  |  |
|  |  | Latest version of Informed consent must be obtained and documented according to GCP guidelines | 1. Patient is submitted to study procedure before the informed consent is obtained (e.g. Condition of patients or emergency situations aggravate timely informed consent process) |  |  |  |  |
|  |  |  | (B) Informed consent is not signed or dated correctly |  |  |  |  |
|  |  |  | (C) Older versions of informed consent documents, that do not include latest protocol amendments are signed by participants |  |  |  |  |
|  | Inclusion/Exclusion | Safety relevant inclusion/exclusion criteria must be considered | Only a subgroup of the population is suitable for the study - safety relevant criteria for inclusion/exclusion |  |  |  |  |
|  | SAE/AE | Causality and medical evaluation of Adverse Events have to be performed thoroughly by qualified staff | (A) Inexperience with study drug (outside authorized indication), drug dose or drug not tested in study population causes misjudgement of Adverse Events (e.g. Serious drug reaction and device effect are not considered for specific study population) |  |  |  |  |
|  |  |  | (B) The potential for an interaction of basic or background therapies, prescribed, recommended or allowed by the protocol is not considered in the evaluation of Adverse Events |  |  |  |  |
|  |  | SAE have to be reported and documented correctly in the required timeframe | Complexity of CRF or missing SOPs for SAE Reporting leads to (A) incorrect documentation and (B) delayed reporting of SAEs |  |  |  |  |
|  | Additional risk element |  |  |  |  |  |  |
| **Overall Study Management** | **Recruitment** | Monthly recruitment should follow the recruitment schedule | (A) Actual participant recruitment is distinctly lower than the estimated recruitment rate |  |  |  |  |
|  |  |  | (B) An alternative treatment competes with participant recruitment |  |  |  |  |
|  |  |  | C) Pre-feasibility assessment of the study recruitment is not based on reliable sources (clinical department activity, pre-screening registry, pilot study) |  |  |  |  |
|  |  | Condition of subject population must be considered recruitment estimations | Difficulty in obtaining informed consent because of the high morbidity of the patient |  |  |  |  |
|  | **Retention** | Loses to follow-up have to be minimized and reasons have to be evaluated | (A) Long follow up times may lead to decreased number of participants (death, unwillingness) |  |  |  |  |
|  |  |  | (B) Follow-up visits cannot be scheduled if prior visits are not reported in a timely manner - study schedule is delayed |  |  |  |  |
|  |  | Condition of subject population must be considered | High drop-out rate because of high numbers of SAE due to severe condition of study population |  |  |  |  |
|  | **Study procedures and endpoint assessments** | Randomization has to take place accurately and within the given Timeframe | (A) Randomization does not take place in the given Timeframe |  |  |  |  |
|  |  |  | B) Randomization ID documentation is prone to error |  |  |  |  |
|  |  | Interim Analysis has to take place within the given Timeframe | Interim Analysis difficult to coordinate (international, multicentre) |  |  |  |  |
|  |  | Procedures for Blinding and unblinding have to be clear | (A) Missing objectivity in the assessment of the primary and main secondary outcomes by unblinded outcome assessors |  |  |  |  |
|  |  |  | (B) No adequate procedures for unblinding in place |  |  |  |  |
|  |  | Complexity of study procedures must be considered and Study conduct must adhere to the protocol procedures | (A) Complexity of study design and treatment schedule increases the risk of non-adherence to the study protocol |  |  |  |  |
|  |  |  | (B) Techniqual requirements - e.g. critical handling of samples/ new assessment tools |  |  |  |  |
|  |  |  | (C) Trial specific knowledge or training required |  |  |  |  |
|  |  | Study Procedures and Conduct must be documented accurately within the given timeframe | (A) Numerous source systems (Electronic and paper source systems) |  |  |  |  |
|  |  |  | (B) Different/Additional data collected in CRF as described in trial protocol/ trial schedule |  |  |  |  |
|  |  |  | (C) Risk for slow data entry in the database |  |  |  |  |
|  | **Endpoint assessment** | Complexity of primary endpoint must be considered | Complex assessment procedure are necessary to obtain the primary endpoint or assessment is not robust (subjective, unblinded, patient-reported) |  |  |  |  |
|  | **Participant Schedule** | Visits/Phone calls must be within the given Timeframe | (A) Time point of visit is critical for the endpoint assessment of the study |  |  |  |  |
|  |  |  | (B) Large number of visits are difficult to organize and coordinate between centres and patients |  |  |  |  |
|  |  | Interventions/medication must be verified and within the given Timeframe | (A) Medication/Intervention at several time points during the day |  |  |  |  |
|  |  |  | (B) Severe condition of patient |  |  |  |  |
|  |  |  | (C) Daily medication not verified by a second person |  |  |  |  |
|  |  | Concomitant therapy should be consistent over the conduct of the study | (A) Heterogeneity of participants morbidities that require different concomitant treatment |  |  |  |  |
|  |  |  | (B) Heterogeneity in procedures between study sites |  |  |  |  |
|  | **Additional risk element** |  |  |  |  |  |  |
| **Device/ Medication Management** | **Storage/ Accountability** | Drug supply must be guaranteed over the whole study period (Production Schedule/ Stock at cites/ Central) | (A) Drug supply channel's validation is not up-to-date, timely supply of medication is endangered. |  |  |  |  |
|  |  |  | (B) Complex IMP shipping process (e.g. location, export conditions, shipping under strict temperature conditions) |  |  |  |  |
|  |  | Drug accountability has to be verified | IMP Handling/preparation/administration has potential for dosing errors, temperature deviations (e.g. self-administration) |  |  |  |  |
|  | **Administration** | Correct IMP administration to the patient must be guaranteed and monitored | (A) Complex IMP handling requirements (e.g. Temperature sensitive, small timeframe till expiration date of the medication (Short shelf-life)) |  |  |  |  |
|  |  |  | (B) Complex IMP administration (e.g. needs preparation with risk of errors/small timeframe between preparation and administration) |  |  |  |  |
|  | **Additional risk element** |  |  |  |  |  |  |
| **Study Data** | **Data Quality** | Study data has to be complete and up to date | (A) Complex assessment procedure are necessary to obtain the primary endpoint or secondary endpoint |  |  |  |  |
|  |  |  | (B) Many data points have to be entered into the CRF |  |  |  |  |
|  |  | Information entered in the electronic CRF must be identical to the source data (Source Data Verification) | (A) No double-data entry implemented |  |  |  |  |
|  |  |  | (B) No source data verification possible |  |  |  |  |
|  |  | Study data must be accurate | Staff is not adequately trained in the generation or documentation of the study data |  |  |  |  |
|  | **Data storage** | Study data must be stored in a safe place. | A) No backup, no audit trail --> loss of data |  |  |  |  |
|  |  |  | B) Source data not locked away --> loss of Source data |  |  |  |  |
|  | **Additional risk element** |  |  |  |  |  |  |
|  |  |  |  |  |  |  |  |
|  |  |  |  |  |  |  |  |

Abbreviations: AE, adverse events; GCP, Good Clinical Practice; IMP, investigational medicinal product; CRF, case report form; SAE, serious adverse events SOPs, standard operating procedures

**Supplementary Figure 1:** Example filter (Panel A), example drop down menu (Panel B)

A) B)


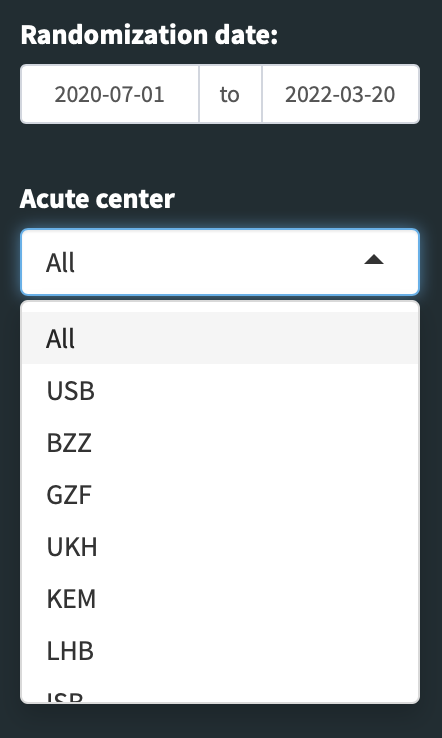

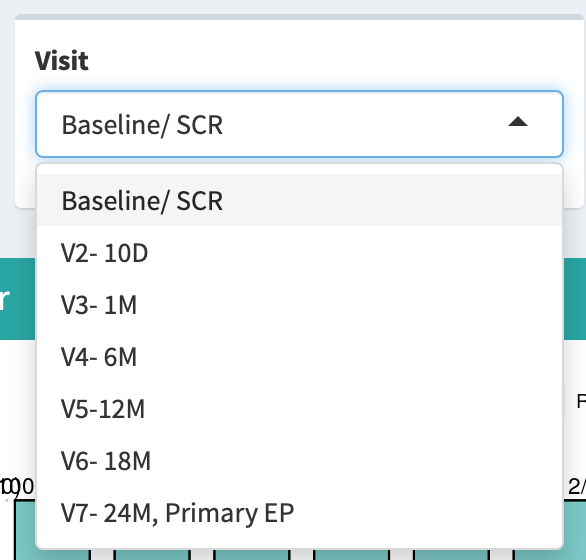


**Supplementary Figure 2:** Recruitment Dashboard – Development (Panel A) and Actual over estimated recruitment (Panel B)

A)


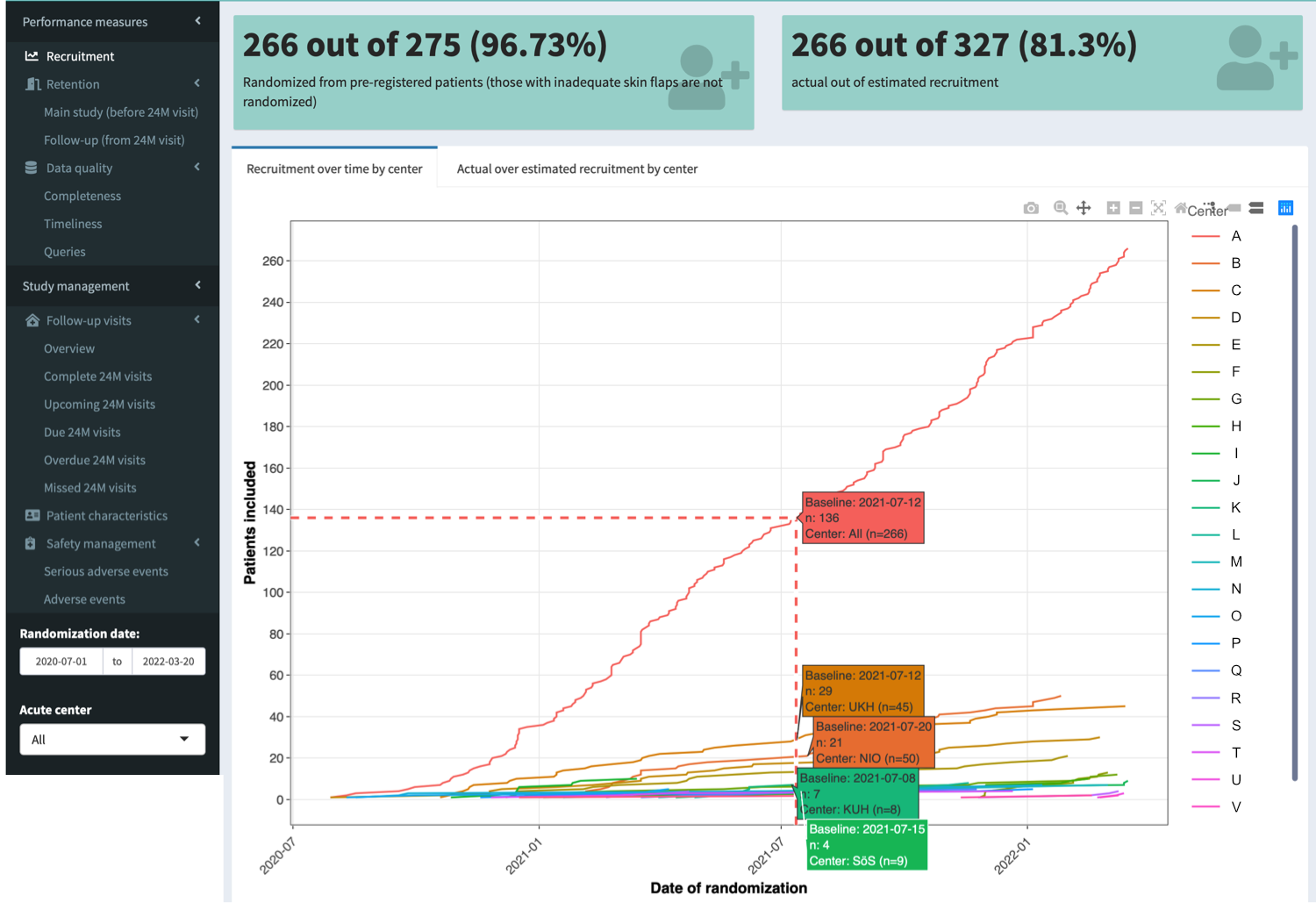


B)


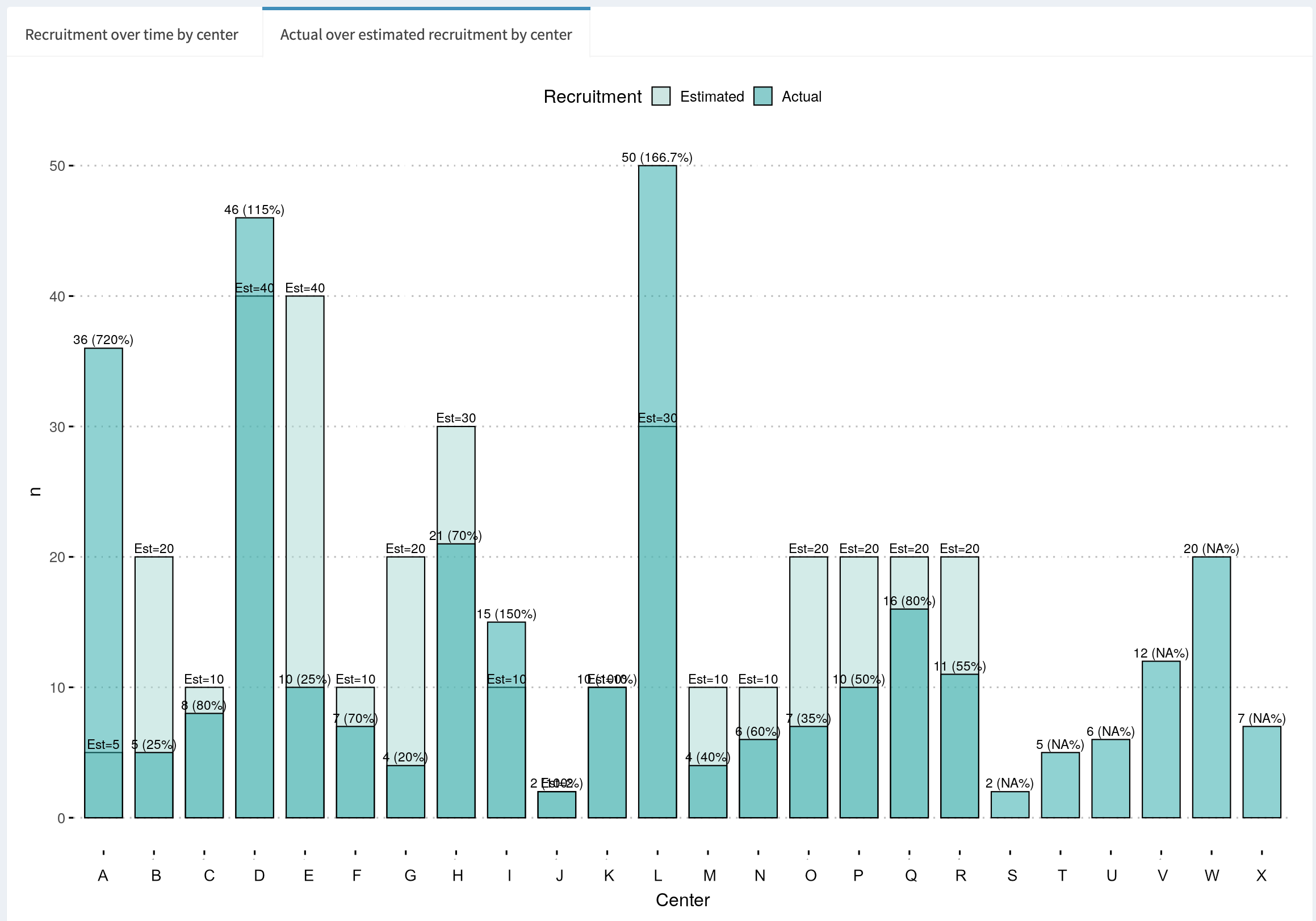


**Supplementary Figure 3:** Retention before Primary Endpoint Assessment


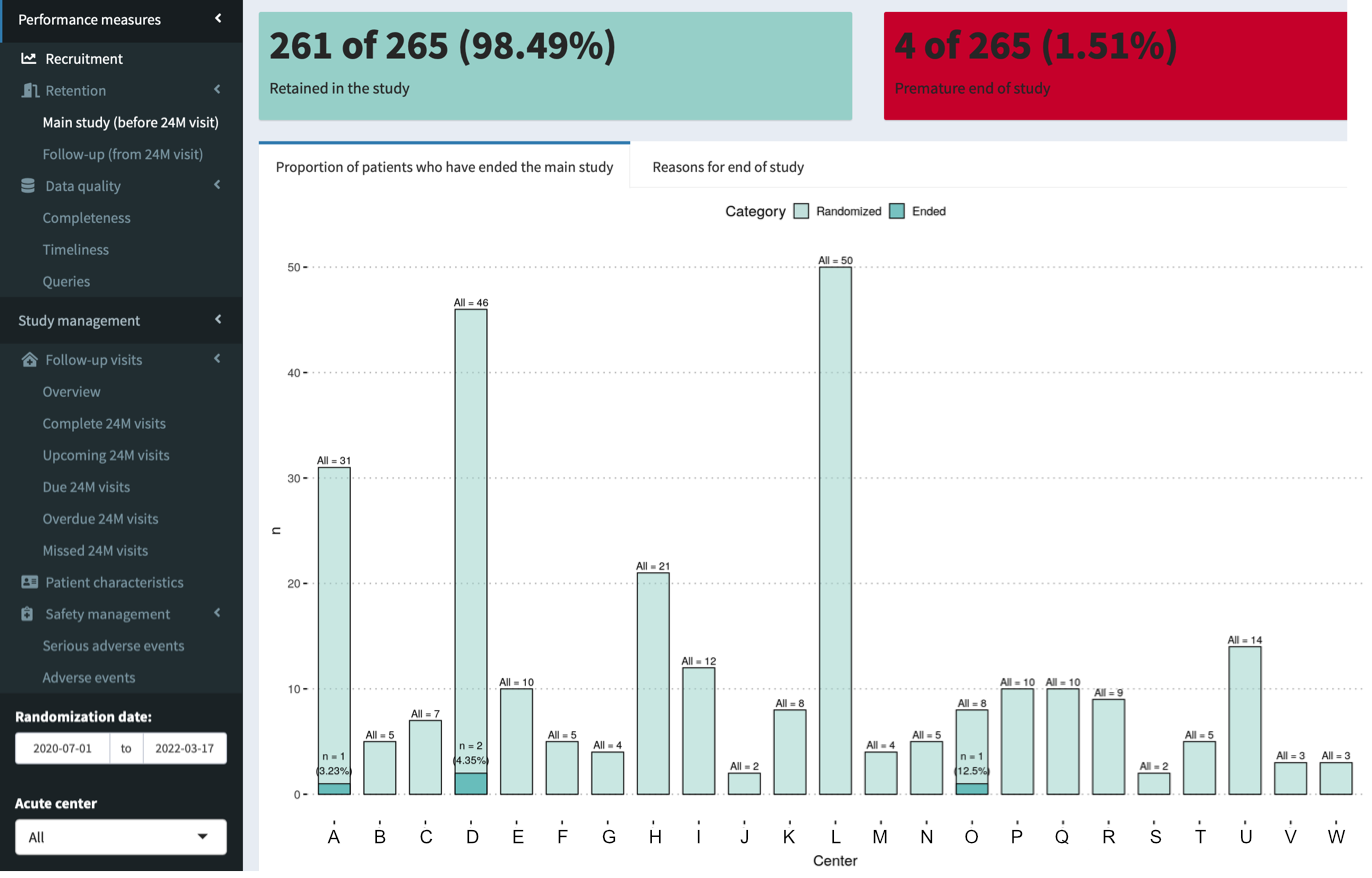


**Supplementary Figure 4:** Data Quality - Completeness of primary endpoint forms


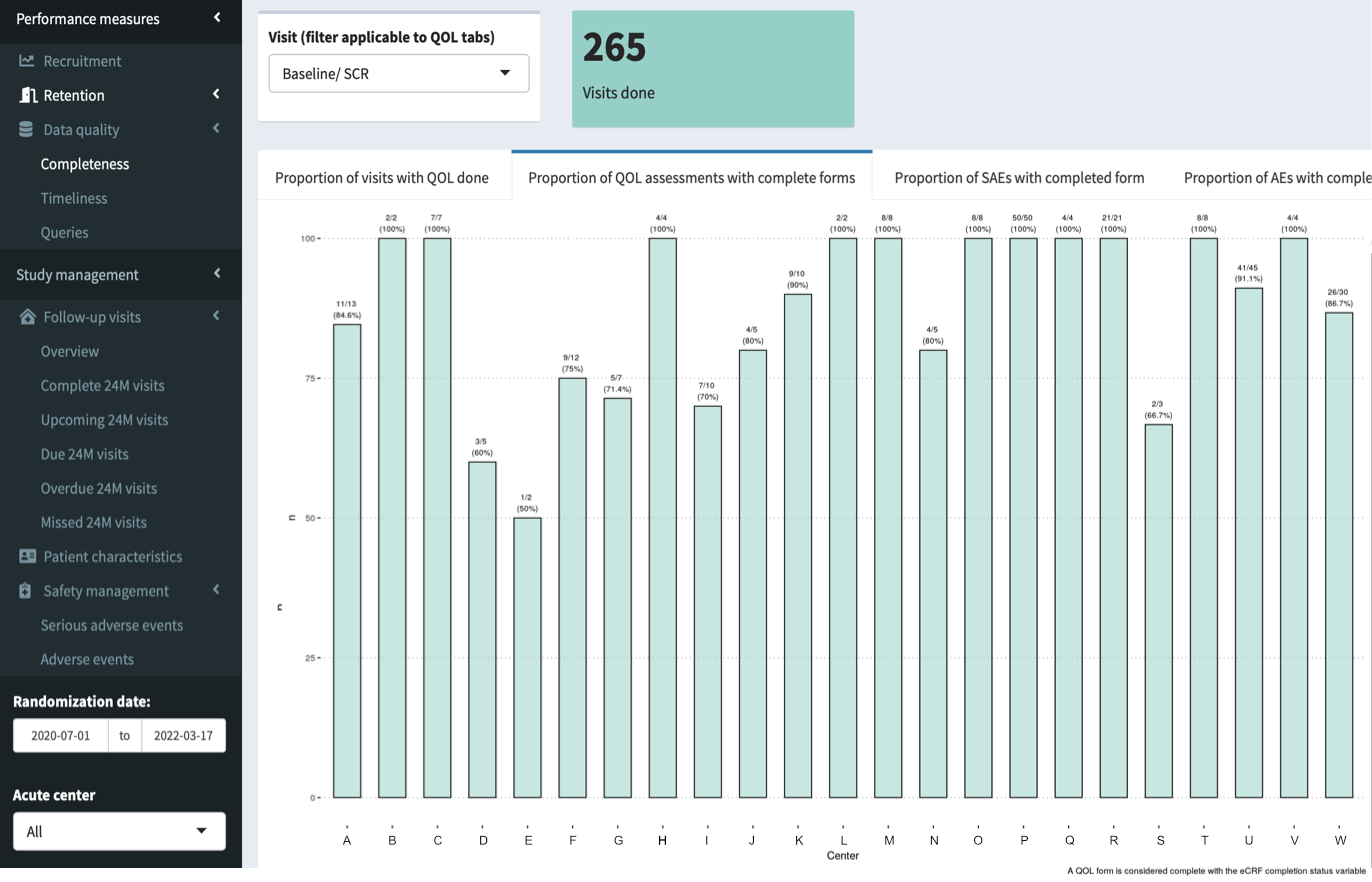


**Supplementary Figure 5:** Status of queries


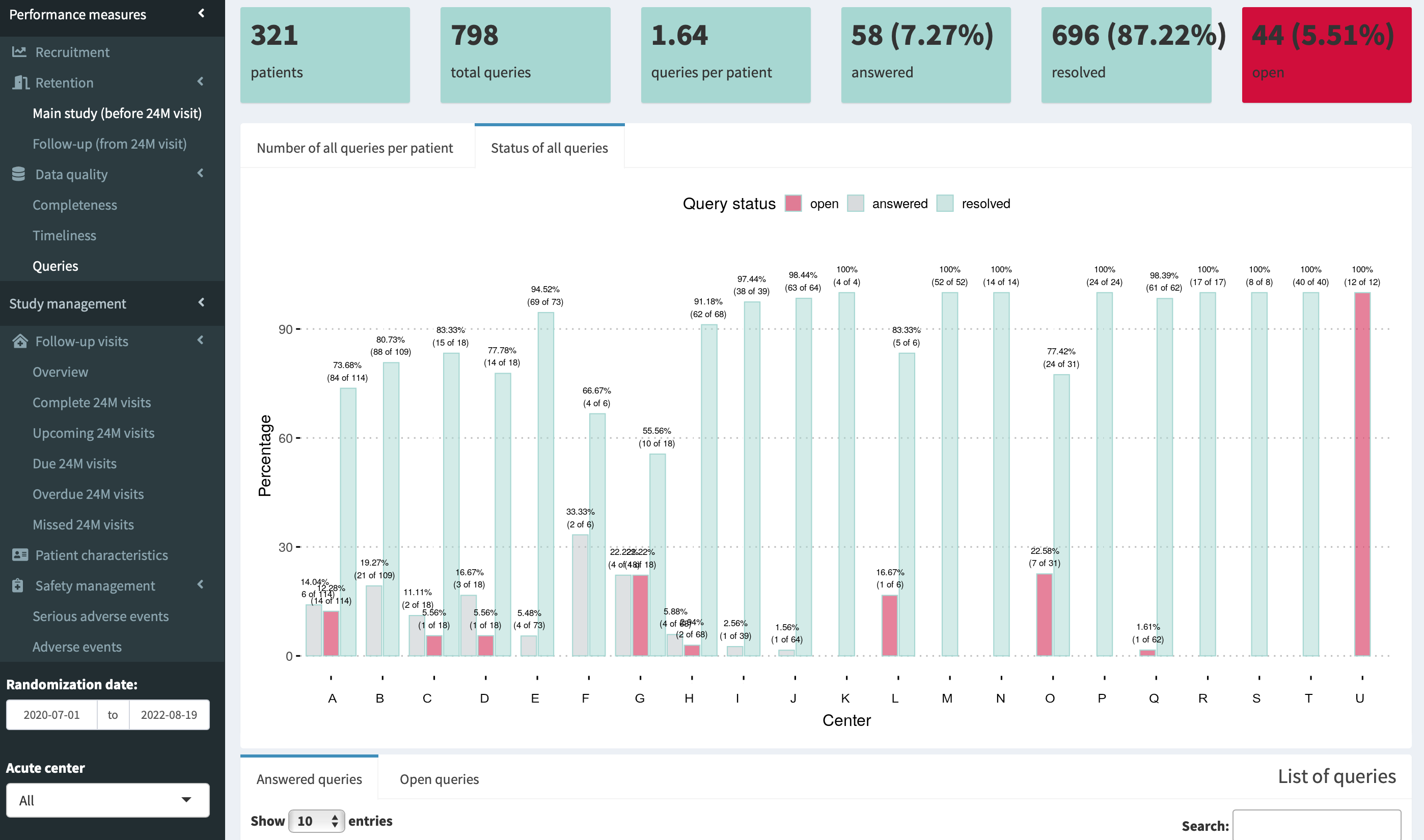


**Supplementary Figure 6:** Example of a query status tab generated from the generic code available on Githup.


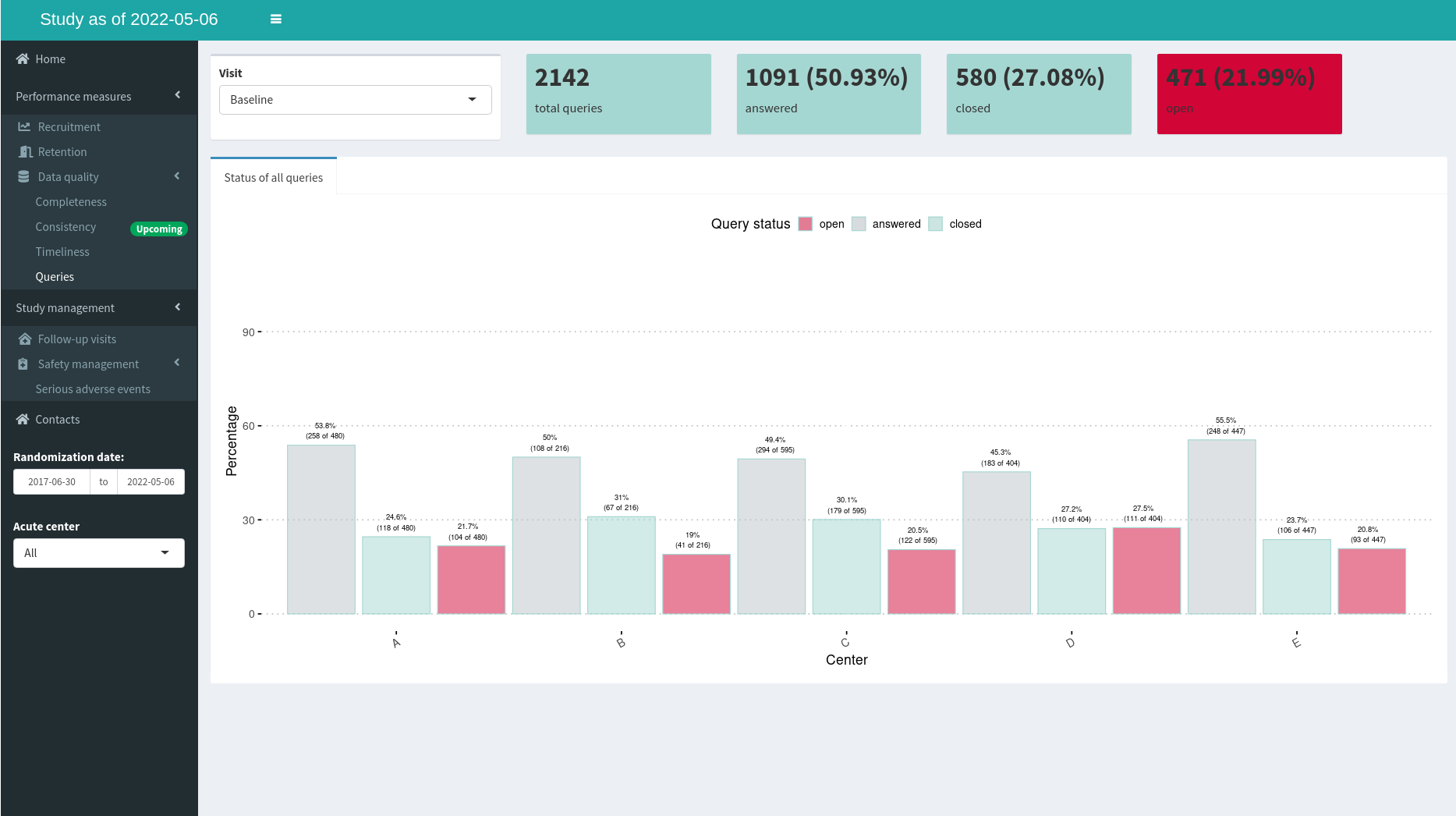


**Supplementary Figure 7:** Example of a safety management tab generated from the generic code available on Githup.


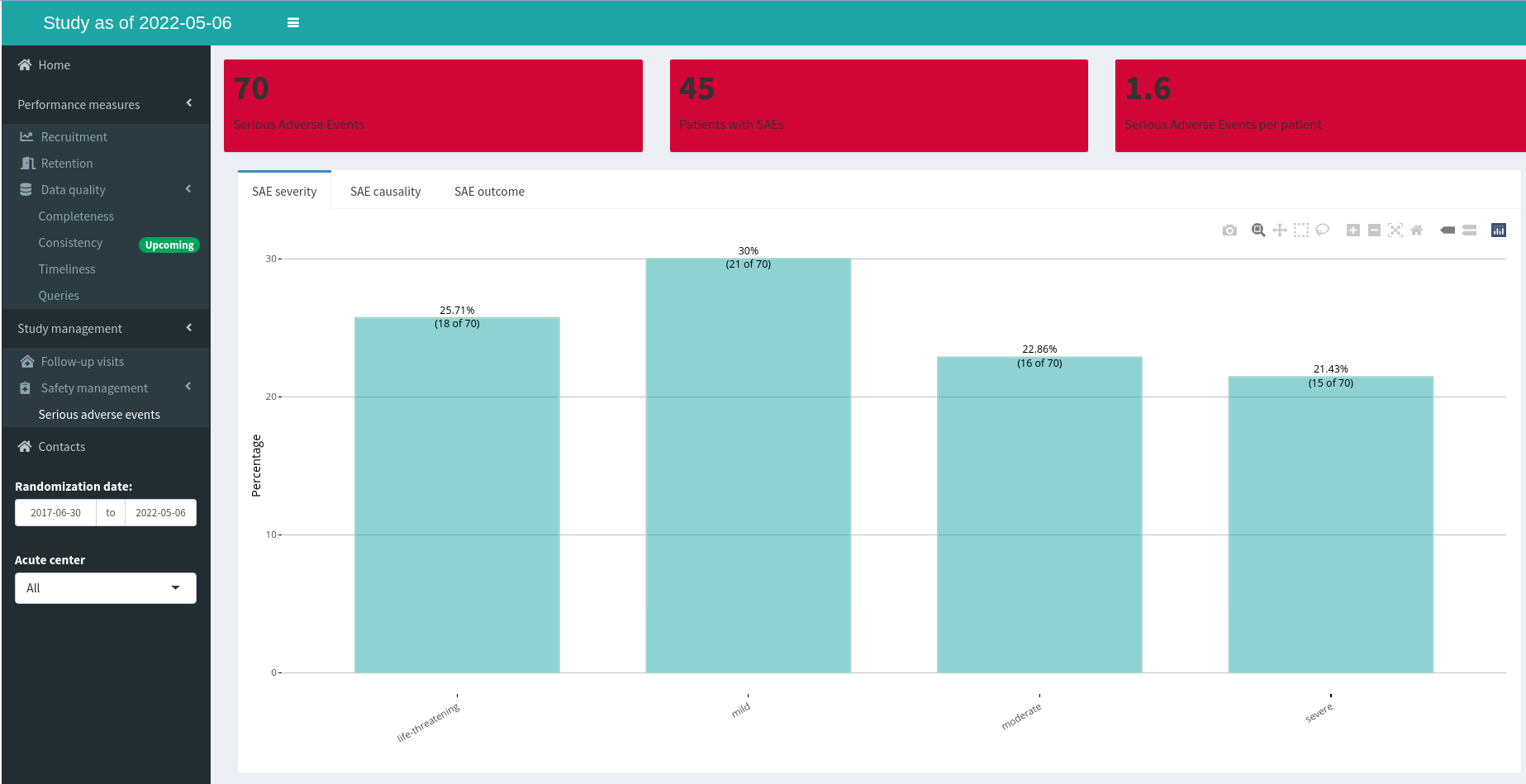


**Supplementary Table 5:** Summary of results from the user testing

| Position | General Feedback | | | Specific feedback | | |
| --- | --- | --- | --- | --- | --- | --- |
|  | Content Risk assessment/dashboard  (Positive and negative comments) | Overall Rating 0-100* | Suggested Applicability | Structure and Design | Additional Tabs | Further suggestions for improvement |
| Trial monitors (n=2) | Dashboard should visualize what the monitoring does not cover. Taking monitoring plan into consideration. | 60-80 | Dashboard for studies with 3 or more sites | Queries –  Resolved – should be colour coded in yellow to resemble database colour code  Spacing numbers/sites | MONITORING tab  Which patients have been monitored – partly monitored is the interesting variable  CONSENT tab – Already program re-consent button into data base in case of amendments – re-consent – overview of which patient signed which version | Check if all visits have been done in the required timeframe- display of delta  Verify entry, informed consent with delegation log |
| Study managers  (n=2) | Preventive – When sites are primed and many problems are solved in advance of the on-site visit–  Increased awareness of data management (what do we need, what is missing)  Improves communication – option for compliments needed  Early awareness of data/ intervention issues  Positive feedback for study coordinator (It is reassuring when the Incomplete box shows 0 patients)  Continuous analysis of SAEs (how many, status)  Overview on data completeness - “Like Central Data cleaning” (which data is missing, identifying key elements of data sheets, number of outcomes analysable) | 65-85 | All studies, depending on the complexity of the intervention and infrastructure | Like driving a car – one red lamp – auto repair shop  All red – not able to drive | VISITS  Include all visits, not just primary outcome visits  ITEM INTERVENTION  (E.g. early discharge needs special attention)  Drug compliance  Premature discontinuation  OUTCOMES  Include secondary outcomes (e.g. recurrent stroke)  HEALTH ECONOMIC OUTCOMES  (E.g. length of stay  Important for Biomarker  SAMPLE MANAGEMENT  Biomarker status and results  (Tracking of sample status)  INFORMED CONSENT  Displaying which protocol and consent versions were effective at which time point in the study | SAE – include narrative of comment field  Login once in secutrial – direct connection to sheet of interest (e.g. SAE form)  App cleaning: differentiate between nothing can be done/ problem that needs to be solved  - Corrected lists or colour coded list  - Better overview  Differentiate between patients died/ withdrawn  Should include more project management aspects – making Excel sheets superfluous  Option for users to enter data and comments to the dashboard) |
| Principal Investigators  (n=2) | Provides an overview over patients with incomplete endpoints, differentiate between outcome measures forever missed and outcomes that may still be possible to extract from source data – list of patients to contact centres  Overview of outcome measures for statistical calculations - 90 % useable outcome  Identify issues that are more prevalent in specific centres.  Very efficient in terms of data completeness  Provides a systematic overview of patient recruitment  Filters are very useful, e.g. show only patients randomized during the COVID pandemic | 95 | All studies  (Dependent on cost benefit relation)  Useful for registers/ cohort studies | Very well designed | Secondary outcomes / other variables / Patient characteristics | Provide basic package for studies to choose the amount and area of support needed |

* Rating from 0 to 100, 100 represents the best evaluation
