# Supplementary material for: Development of a risk-tailored approach and dashboard for efficient management and monitoring of investigator-initiated trials
Source: BMC Med Res Methodol. 2023 Apr 5;23:84. doi: 10.1186/s12874-023-01902-y (PMC10074803; doi:10.1186/s12874-023-01902-y)
Supplement: Supplementary file 2 — Supplementary Material 2 [file 12874_2023_1902_MOESM2_ESM.doc]

**Risk-tailored Approach for Efficient Management and Monitoring of Investigator-Initiated Trials**

MANUAL

List of Contents

[Intention 1](#__RefHeading___Toc25743985)

[Requirements and Basic Elements 2](#__RefHeading___Toc25743986)

[1st step: Identification of Risks 2](#__RefHeading___Toc25743987)

[2nd step: Analysis of Risks 5](#__RefHeading___Toc25743988)

[3rd step: Development of Control Pathways 8](#__RefHeading___Toc25743989)

[4th step: Implementation of Control Pathways 8](#__RefHeading___Toc25743990)

# Intention

The main focus of the risk-tailored approach for efficient trial management is to support the conduct of clinical trials by providing a continuous oversight on the correct implementation, progress and accuracy of the most important elements of a clinical trial. We define an asset of a clinical trial as a standard requirement that provides the basis for safety and accuracy of a clinical trial. An example for an ASSET in a clinical trial could be the condition that the recruitment of participants should follow the expected recruitment rate or that visits must be scheduled and take place in the required timeframe.

In the first step, the developed risk assessment guide supports the Principal Investigator (PI) or project manager in the identification of the critical elements of an individual clinical trial. Factors that potentially impact the likelihood or severity of the risk have to be considered in the risk analysis step. Once study elements that are potentially at risk have been identified, CONTROL PATHWAYS are developed and implemented in order to provide a continuous oversight of these elements. The continuous overview of these elements supports the trial management and enables preventive measures and early interference. Oversight on data completeness, query status, SAE reporting and Informed consent status can also support and guide the on-site monitoring by prioritizing sites, and participants.

The risk-tailored approach consists of 4 steps, which may be repeated or updated throughout the study conduct:

1. Identification of study-specific risks
2. Analysis of the risks
3. Development of control pathways
4. Implementation of control pathways

# Requirements and Basic Elements

In order to perform the risk assessment of a particular study, the following resources are required:

- Study Protocol
- Case Report Form (CRF) structure
- Established contact to responsible data manager of the study
- Overview of staff assigned to the study (e.g. Delegation Log, Curriculum Vitae and research experience of study staff)
- Information on the planned and actual budget of the study (Feasibility report)
- Expected recruitment for all participating centres
- Investigational Medicinal Product (IMP) information e.g. information about the handling of the IMP in terms of storage, transport and expiration date, information on supply chain (more than one supplier), organization of IMP distribution to participating centres.
- Established contact with the principal investigator

# 1st step: Identification of study-specific risks

Study elements are categorized in 4 domains, which represent different aspects of the clinical trial conduct:

1. Participant Safety and Rights
2. Overall Study Management (Study procedures, Participant Schedule)
3. Device/ Medication Management
4. Study Data

**Table 1: Risk elements of the 4 domains.**

| Domain | Risk Elements |
| --- | --- |
| Participant Safety and Rights | Informed consent  AE/SAE reporting and documentation  Inclusion/exclusion |
| Overall Study Management | Recruitment  Retention  Study procedures and endpoint assessment (e.g. bio sampling, imaging quality)  Participant schedule (e.g. timeframe of visits)  AE/SAE management |
| Device/ Medication Management | Administration  Accountability/ storage |
| Study Data | Data quality – completeness, consistency, timeliness  Documentation/ storage |

Abbreviations: SAE, Serious Adverse Events

These domains contain risk elements covering most important elements of trial conduct. In order to identify the CRITICAL ASSETS of a trial, essential information has to be extracted and collected from the listed sources. To evaluate the applicability of an ASSET, the structure of the risk assessment guide includes one or more possibly applicable RISK SCENARIOS that endanger the ASSET or its accuracy (Example provided in **Figure 1**). In the process of identifying study specific risks, all listed ASSETS and the corresponding RISK SCENARIOS in the template are being evaluated with respect to the particular clinical trial. Every RISK SCENARIO that might apply to this trial should be marked (example shown in **Figure 2**).

**Asset:**  Value of standard requirements that provide the basis for safety and accuracy of a clinical trial

**Risk:** Definition of risk including the source of the risk (Risk scenario)


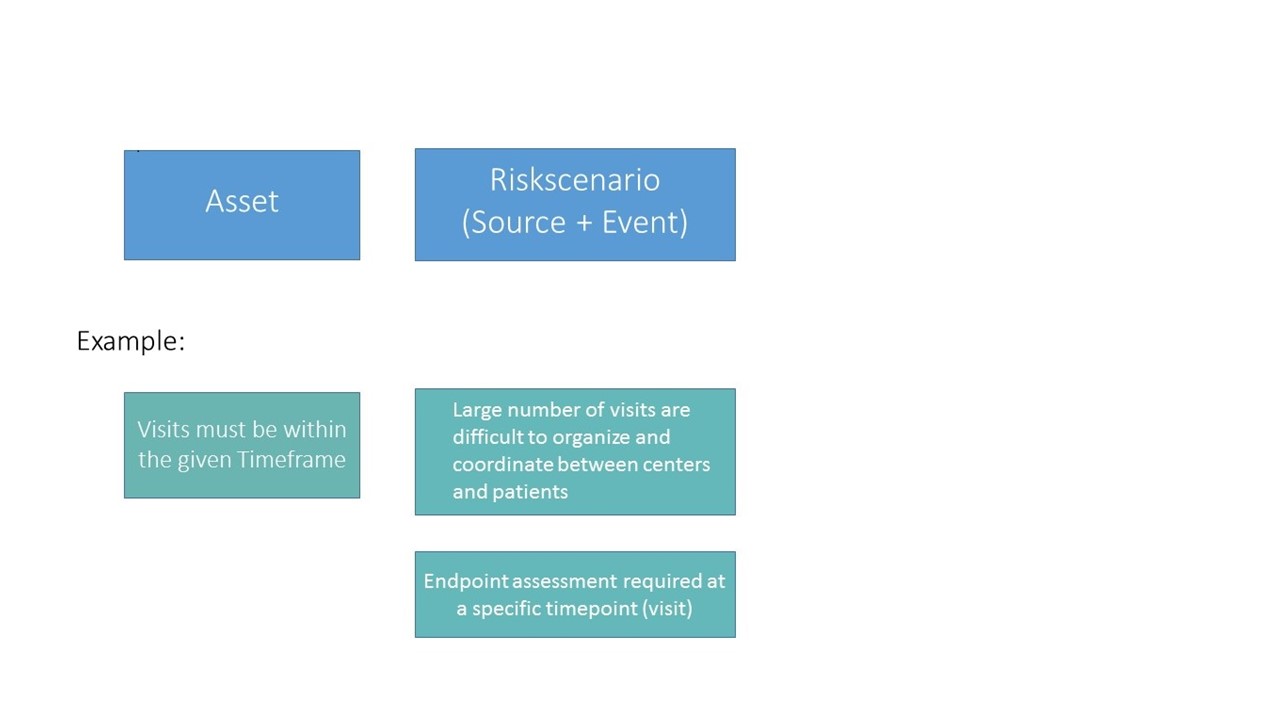


Fig 1: Example of RISK SCENARIOS for an ASSET

| Domain | Risk element | Asset | Risk scenario | Facilitator* | Likely | Critical | Rationale for Rating |
| --- | --- | --- | --- | --- | --- | --- | --- |
| **Overall Study Management** | **Participant Schedule** | Visits/Phone calls must be within the given Timeframe | (A) Time point of visit is critical for the endpoint assessment of the study |  |  |  |  |
|  |  |  | (B) Large number of visits are difficult to organize and coordinate between centres and patients |  |  |  |  |

**Fig 2: Structure of Risk assessment –Example of an asset from the Overall Study Management domain.**

* Check for applicability of one of the Facilitators for the identified risk

(1) Experience with similar study or pilot study conducted - Similar study was successfully conducted in the same setting (similar infrastructure, Procedures, Intervention); (2) Well-trained, experienced, and dedicated principal investigators and study staff present Number of successful clinical trials completed by the PI/ Number of clinical trials supported by Study Nurse; (3) Adequate Budget – Feasibility report available.

# 2nd step: Analysis of risks

In order to estimate the risk for a specific scenario/asset, further information on the study may have to be obtained from the PI or study manager, e.g. information on the infrastructure of participating centres, actual budget etc., and considered in the analysis. The impact of the following three FACILITATORS will be assessed and considered in the LIKELIHOOD rating of each trial-specific RISK SCENARIO.

**Facilitators:**

1. Experience with similar study or pilot study conducted

- Similar study was successfully conducted in the same setting (similar infrastructure, Procedures, Intervention)

1. Well-trained, experienced, and dedicated principal investigators and study staff present

Define experience:

- Number of successful clinical trials completed by the PI
- Number of clinical trials previously supported by designated study staff

1. Adequate budget

- Check actual budget against estimations in the budget plan or budget needs as judged by an experienced trial manager (actual budget opulent/sufficient vs. low/tight)

**Likelihood:** The evaluation of the likelihood of a risk scenario for most of the assets is highly influenced by the applicability of the three facilitators. If the budget of the study is adequate and as planned as well as well trained, experienced staff is present or a similar study has already been conducted in the same setting, the likelihood for the risk scenario decreases.

Likelihood description: Likely (including possible, almost certain) vs. Unlikely (including rare)

The estimation of the likelihood must be updated regularly during trial conduct, e.g. in case of staff fluctuations or changes in the funding/budget situation

The SEVERITY will be mainly influenced by the CONSEQUENCES of the specific RISK SCENARIO on the overall study.

**Consequence:** The impact of the risk when the critical asset is not met

**Severity:** Assessing the question: How critical is the consequence for the overall study conduct, study outcome, and patients’ rights and safety. In order to categorize consequences and enable an analysis of the risk, critical and non-critical consequences are defined according to the classification of Good Clinical Practice /GCP) findings described in “Procedure for reporting of GCP inspections requested by the Committee for Medicinal Products for Human Use (CHMP)” by the European Medicines Agency in 2017.

(Available under https://www.ema.europa.eu/en/documents/regulatory-procedural-guideline/ins-gcp-4-procedure-reporting-good-clinical-practice-inspections-requested-chmp_en.pdf).

Definition for critical: Conditions, practices, or processes that endanger the rights, safety or well being of the participants or the protocol-conform collection of outcome data.

Possible consequences: Rejection of data and/or legal action is required

Definition for non-critical: Conditions, practices, or processes that deviate from the planned conduct, but are not expected to endanger the safety or well being of the participants or the protocol-conform collection of outcome data.

Possible consequences: Data might be rejected or the duration of the study might be extended. Non-critical applicable risk scenarios still indicate the need for improvement of conditions, practices and processes.

For each applicable ASSET, a rating will be performed for the SEVERITY and LIKELIHOOD based on the CONSEQUENCES of the RISK SCENARIO and the applicability of FACILITATORS for this ASSET. Other factors such as experience with a similar intervention or process might also influence the rating; therefore it is important to provide a short rationale for the rating. All assets that are rated “likely” and/or “critical” will then be included in the development and implementation of control pathways. Examples of the evaluation of assets are provided in **Table 2**.

**Table 2: Example of a risk assessment and risk analysis** of four different assets

| Asset | Risk Scenario | Facilitator | Likely | Critical | Rationale for Rating |
| --- | --- | --- | --- | --- | --- |
| Study data has to be complete and up to date | Incomplete Data and incorrect Data transfer - Compromising the assessment of the primary and secondary endpoints | None applicable | ✓✓  x |  | Endpoint is assessed at Rehabilitation centre, slow or incomplete data transfer (input of many data points into CRF) is likely |
| Monthly recruitment should follow the recruitment schedule | Insufficient participant recruitment - Extended study duration and increased costs | Inexperienced centres included | / ✓✓  x | / ✓✓  x | Many smaller inexperienced centres and a competing alternative treatment |
| Visits/phone calls must happen within the given timeframe | Impacts the analysis of the primary endpoint | No study nurse present | / ✓✓  x | / ✓✓  x | Primary endpoint is assessed at 3-month visit, which takes place at the rehabilitation centres (no experienced staff present). |
| Complexity of primary endpoint must be considered | Validity of primary outcome is low | Second blinded assessment by experienced PI | / ✓✓  x | / ✓✓  x | Endpoint assessment is complex but staff is specially trained and there is a second blinded assessment by the PI. |

Abbreviations: PI, Principal Investigator, CRF, Case Report Form.

# 3rd step: Development of Control Pathways

All ASSETS and the corresponding RISK SCENARIOS will be included in the development of PATHWAYS. PATHWAYS are a set of operations applied to the data set (collected through the CRF) and supporting information (expected recruitment, shipping information etc.) and will generate an OUTPUT that will be visualized (Graph/color-coded panels) for clinical monitors and study staff in the dashboard application. An example for a PATHWAY is provided in **Table 3**.

**Table 3**: Example of pathways for Likely and/or Critical Risk scenarios

| Risk scenario | Pathway | Dashboard ID |
| --- | --- | --- |
| **Time point of visit is critical for the endpoint assessment of the study** | Calculation of visit plan based on randomization date of patient -> Continuous overview of upcoming visits (possibility for reminders)Due visits (+/- 14 days) – number of patients (per centre) and list of patients Overdue visits (+/- x days - depending on the timeframe stated in the study protocol) - number of patients (per centre) and list of patients  Missed visits (out of acceptable timeframe) – number of patients (per centre) and list of patients | FOLLOW-UP VISITS |

# 4th step: Implementation of Control Pathways

Dashboard Approach:

Each pathway is assigned to a DASHBOARD ID/TAB. The visualization of the control pathway OUTPUT is accessible under this ID/TAB in the dashboard. The OUTPUT is based on daily data exports from the trial database. Examples of the visualized OUTPUT in the dashboard are provided in **Figure 2** and **Figure 3**. A basic structure of the pathways of generic Tabs is provided in form of modules (mod) in the Githup environment (<https://github.com/CTU-Basel/viewTrial>) and listed in **Table 4**. The template structures can be downloaded and adapted to the specific database format and structure of exported tables containing relevant variables.


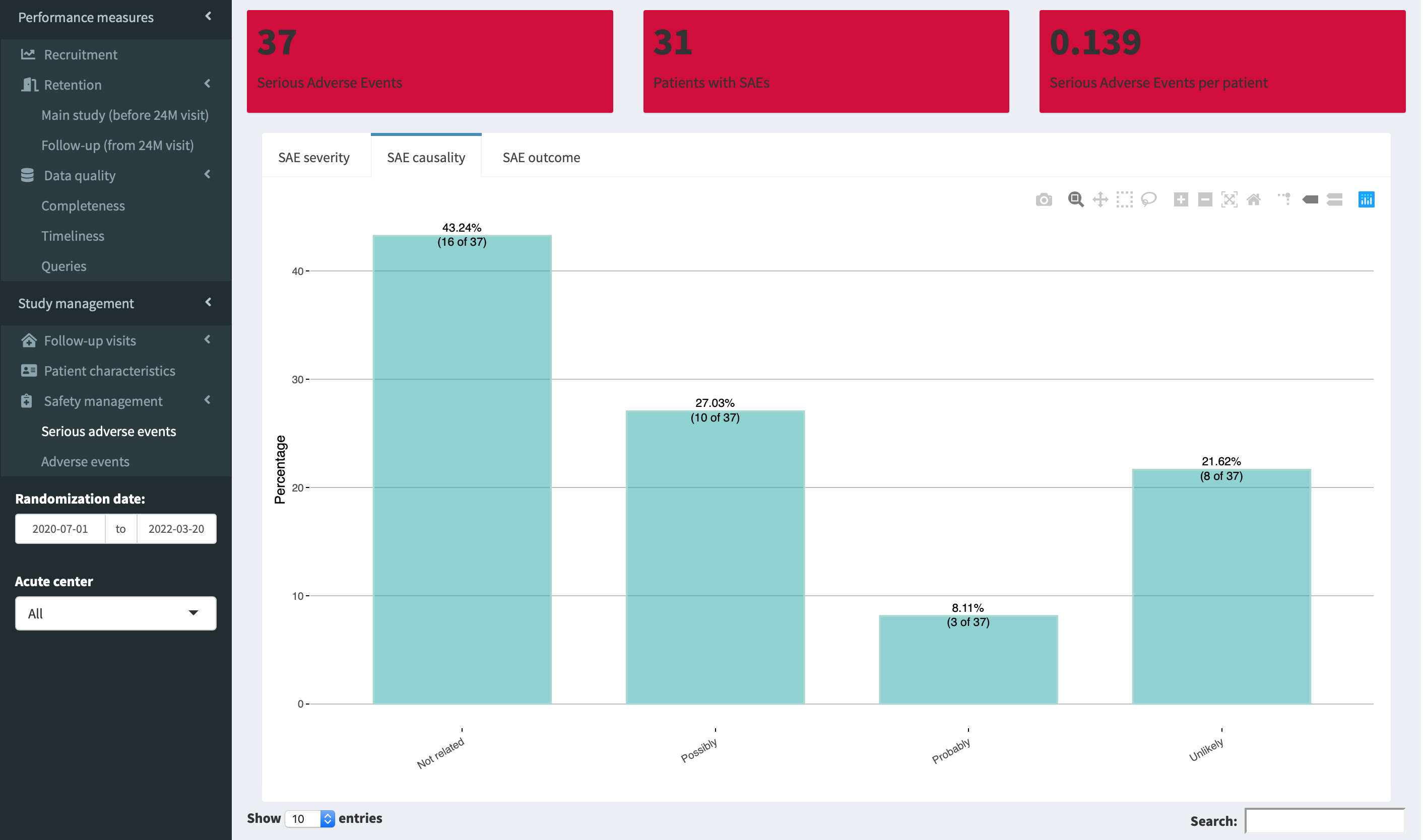
/

**Fig 2: Example of the visualization control pathway OUTPUT in the Safety management TAB**


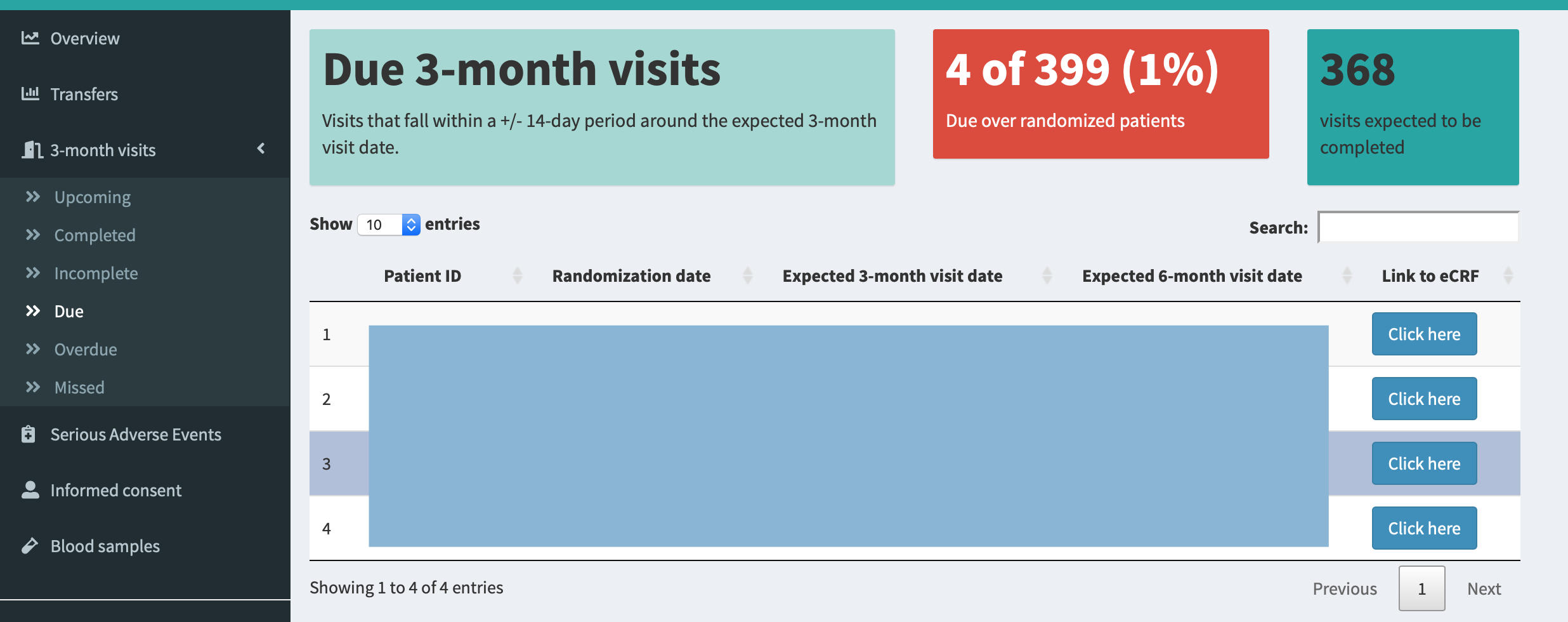
/

**Fig 3: Example of the visualization control pathway OUTPUT in the Visits Tab**

**Table 4**: List of Dashboard ID/Tabs of the generic dashboard template

| **TAB** | **Basic pathways structure (modules, mod) provided in the template** |
| --- | --- |
| **Recruitment** | Mod_recruitment provides a recruitment plot together with two information boxes |
| **Recruitment** | Mod_retention provides details on number and reason of loss to follow up |
| **Data quality** | Mod_completeness provides an example of how data completeness might be shown  Mod_timeliness provides an example of how time between events and their entry into the database might be shown  Mod_queries provides an example of how number of queries and query status might be shown |
| **Follow-up visits** | Mod_fup provides an example of how tracking of participant progress through a trial might be shown |
| **Safety management** | Mod_sae shows counts of SAE and characteristics of reported SAEs |
